# BEES-HAUS preventing urethral stricture recurrence by restoring the integrity of urothelium and its further simplified version, the BHES-HAUS

**DOI:** 10.3389/fbioe.2025.1687741

**Published:** 2025-11-27

**Authors:** Akio Horiguchi, Surya Prakash Vaddi, Senthilkumar Rajappa, Senthilkumar Preethy, Samuel J. K. Abraham

**Affiliations:** 1 Division of Reconstruction, Center for Trauma, Burn and Tactical Medicine, National Defense Medical College Hospital, Tokorozawa/Saitama, Japan; 2 Department of Urology, Surya Kidney Centre, Hyderabad, India; 3 Department of Urology, Kamineni Academy of Medical Sciences and Research Centre, Hyderabad, India; 4 Antony- Xavier Interdisciplinary Scholastics (AXIS), GN Corporation Co. Ltd., Kofu, Japan; 5 Surya Akio Horiguchi Lab for Tissue Engineering (SALT), Soul Synergy, Phoenix, Mauritius; 6 The Fujio-Eiji Academic Terrain (FEAT), Nichi-In Centre for Regenerative Medicine (NCRM), Chennai, Tamil Nadu, India; 7 Centre for Advancing Clinical Research (CACR), University of Yamanashi - School of Medicine, Chuo, Japan; 8 Mary-Yoshio Translational Hexagon (MYTH), Nichi-In Centre for Regenerative Medicine (NCRM), Chennai, India; 9 Levy-Jurgen Transdisciplinary Exploratory (LJTE), Global Niche Corp., Wilmington, DE, United States

**Keywords:** urethral stricture, buccal mucosa, BEES-HAUS, BHES-HAUS, urothelium

## Abstract

**Figure:**
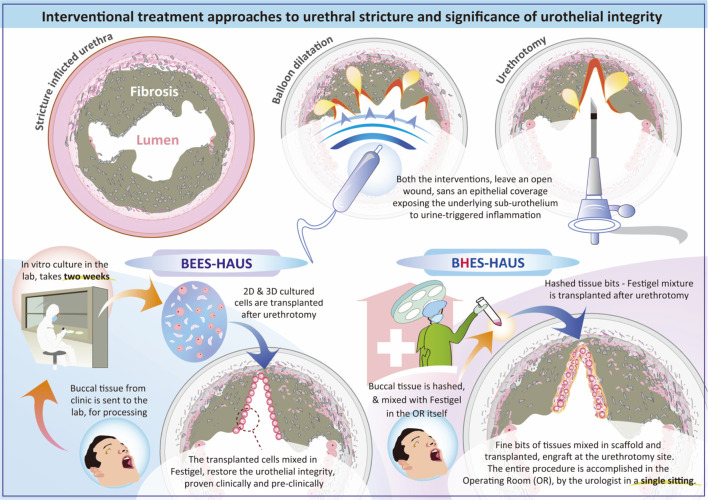
Diagram illustrating interventional treatment approaches for urethral stricture, emphasizing urothelial integrity in preventing stricture recurrence. It compares procedures such as balloon dilatation and urethrotomy which leave the sub-urothelium exposed to urine leading to inflammation to that of procedures such as BEES-HAUS in which transplanted buccal mucosal cells and in BHES-HAUS transplanted buccal tissue bits, both mixed with Festigel, engraft and restore the urothelial integrity. BEES-HAUS involves a two-week in vitro culture, while BHES-HAUS processes tissue in the operating room in single sitting. Both methods aim for effective urethral treatment using autologous buccal mucosal epithelium.

Diagram illustrating interventional treatment approaches for urethral stricture, emphasizing urothelial integrity in preventing stricture recurrence. It compares procedures such as balloon dilatation and urethrotomy which leave the sub-urothelium exposed to urine leading to inflammation to that of procedures such as BEES-HAUS in which transplanted buccal mucosal cells and in BHES-HAUS transplanted buccal tissue bits, both mixed with Festigel, engraft and restore the urothelial integrity. BEES-HAUS involves a two-week in vitro culture, while BHES-HAUS processes tissue in the operating room in single sitting. Both methods aim for effective urethral treatment using autologous buccal mucosal epithelium.

## Introduction

Urethral stricture disease (USD) is a persistent, distressing condition that leads to considerable urinary difficulties and sexual complications (Zheng et al., 2019). Urethral stricture is the narrowing of the urethral lumen caused by fibrotic changes arising from congenital or acquired pathological conditions, with an estimated prevalence of about 0.6%. USD denotes the progressive scarring process that involves the urethral epithelium or the spongy erectile tissue of the corpus spongiosum (spongiofibrosis) (Noureldin et al., 2021). While USD may remain asymptomatic in its early stages, progressive narrowing of the lumen eventually leads to significant voiding difficulties. The causes of urethral strictures are broadly categorized into five groups: idiopathic, iatrogenic, infections, inflammatory, and traumatic (Grimes et al., 2019). Among these, idiopathic and iatrogenic etiologies are the most frequent, each contributing to approximately one-third of cases. Traumatic origins account for about 19%, while inflammatory causes make up around 15% (Grimes et al., 2019).

The pathophysiology of urethral stricture disease is primarily driven by fibrosis. Evidence suggests that patients with strictures exhibit alterations in the extracellular matrix (ECM) glycosaminoglycan profile compared to healthy individuals, with reduced hyaluronic acid and increased dermatan sulfate concentrations. These findings indicate that stricture segments represent a later stage of wound healing and point toward prior urethral injury as a key etiological factor. Moreover, chronic inflammation plays a central role, as fibrosis represents the end stage of persistent inflammatory processes and is largely irreversible (Abdeen et al., 2025). Injury to the urethral epithelium either through urine extravasation into the corpus spongiosum or direct trauma triggers inflammation and subsequent fibrotic remodeling of the spongiosum. The accumulation and contraction of fibrotic tissue progressively narrow the urethral lumen. Additionally, epithelial metaplasia to stratified squamous epithelium increases susceptibility to mechanical stress and microtrauma. This heightened vulnerability predisposes to mucosal tears, further urinary leakage, and perpetuation of fibrosis. Consequently, a vicious cycle of epithelial damage, inflammation, and fibrotic remodeling results in progressive urethral narrowing and recurrent stricture formation (Abdeen et al., 2025).

### Management approaches to USD

In the absence of complications, the primary goal of USD management is symptom relief. Treatment decisions are guided by symptom severity, stricture location, length, and patient preference. In healthy young men, the normal peak urinary flow rate (Q-max) is > 15 mL/s, whereas most patients with strictures present with reduced flow rates (<12 mL/s). Those with flow rates between 10 and 15 mL/s are often asymptomatic and typically do not require intervention, provided there is no evidence of bladder wall thickening or incomplete emptying. A peak flow rate of 5–10 mL/s, however, is usually associated with obstructive symptoms and potential complications (Abdeen et al., 2025). In cases of acute urinary retention or related complications, urgent interventions include urethral dilation, cystoscopy, direct vision internal urethrotomy (DVIU), or suprapubic cystostomy. Broadly, treatment strategies fall into two categories which are endoscopic approaches such as urethral dilatation and DVIU and open surgical procedures such as stricture excision and anastomotic urethroplasty, substitution urethroplasty and diversion like perineal urethrostomy. Urethral dilatation is performed to gradually widen the narrowed urethra caused by stricture which can be achieved using two main techniques (Abdeen et al., 2025), bougie or sound dilatation wherein a series of progressively larger, lubricated rods (bougies or sounds) are gently passed into the urethra to enlarge the lumen and identify sites of obstruction or balloon dilatation where an inflatable balloon catheter is introduced under urethroscopic guidance, usually over a flexible guidewire. Once positioned across the stricture, the balloon is inflated to expand the narrowed segment, providing a less traumatic alternative to traditional dilatation methods (Li X. et al., 2024). Next is DVIU, the most common first-line option for short (<2 cm) untreated bulbar strictures which involves a transurethral incision at the 12 o’clock position to release the fibrotic segment, allowing secondary healing and lumen expansion. Despite being effective in select patients, recurrence rates remain high, reaching up to 65% within 3 years. Recent studies suggest adjunctive treatments may improve outcomes. For instance, intralesional botulinum toxin injection administered during DVIU has been shown in a randomized, double-blind trial to enhance prognosis and reduce recurrence (Abdeen et al., 2025). Similarly, paclitaxel-coated balloon dilation combined with DVIU has demonstrated superior results in recurrent bulbar strictures <3 cm compared to DVIU alone (Virasoro et al., 2020). Paclitaxel, widely used in vascular interventions for its anti-inflammatory and anti-proliferative effects, provides localized drug delivery to the strictured segment, inhibiting scar tissue regrowth and markedly lowering recurrence rates (Abdeen et al., 2025; Virasoro et al., 2020).

Urethroplasty is considered as the gold standard for managing urethral stricture and stenosis, with both excisional urethroplasty and graft-based techniques showing superior long-term success compared to other modalities. Excision and primary anastomosis (EPA), which involves removing the fibrotic segment and rejoining the healthy urethral ends, is regarded as the optimal treatment for short bulbar strictures regardless of cause or prior interventions (Gallegos and Santucci, 2016).

However, a limitation of all the endoscopic procedures is the lack of epithelial coverage, leaving subepithelial tissue exposed to urine, which may promote infection and myofibroblast proliferation leading to fibrosis, thereby contributing to recurrence (Abbas et al., 2019; Hirano et al., 2023). Avoidance of urine leakage is a critical factor in preventing stricture formation, particularly given the highly cytotoxic nature of urine. In the normal urethra, the barrier function of the urothelium is maintained through three key components: uroplakin proteins within the apical cell membrane, tight junctions between superficial umbrella cells, and a protective layer of glycosaminoglycans (GAGs) and proteoglycans coating the umbrella cell surface. Thus, proper formation and regeneration of the urothelium are essential to maintain this barrier, as disruption can predispose to urethral stricture development. Furthermore, an intact urothelium plays an important role in preventing detrusor muscle overactivity, inflammation, and fibrosis within the urinary tract (Abbas et al., 2019).

This drawback of lack of epithelial coverage is addressed in open surgical procedures, where grafts or flaps provide epithelial lining and structural reinforcement, particularly useful for long or anatomically complex strictures such as those in the penile urethra (Carr et al., 1997). Historically, penile or scrotal skin flaps were used but had failure rates of 20%–30%, while split-thickness skin grafts (STSGs) achieved around 80% success (Carr et al., 1997) but required multiple stages and carried donor-site morbidity; bladder mucosa has also been used but with moderate failure rates and the disadvantage of invasive harvesting. The introduction of buccal mucosal grafts (BMGs) in the 1990s transformed urethral reconstruction due to their robust, non-keratinized stratified squamous epithelium, similarity to urethral tissue, resistance to infection, thick elastic epithelium, thin lamina propria, and abundant availability with minimal donor-site morbidity (Foreman et al., 2023). However, the use of buccal mucosal grafts is not without limitations, as the restricted availability of autologous tissue and donor site morbidity remain significant clinical challenges. Patients undergoing buccal tissue harvest may develop scarring, contracture, persistent pain, numbness, or parotid duct injury, with reported oral complication rates ranging from 3% to 4% (Abbas et al., 2019). Moreover, buccal mucosa urethroplasty is generally reserved for the later stages of disease when less invasive options are no longer feasible. The procedure is also associated with higher costs and prolonged hospital stay compared to other reconstructive techniques. These limitations have driven growing interest in alternative strategies, particularly tissue-engineered grafts, which aim to provide readily available, biocompatible substitutes that overcome donor site morbidity while offering durable long-term outcomes (Abbas et al., 2019).

### Unique advantages of BEES-HAUS

Since urine exerts harmful effects on the cellular components of a tissue-engineered urethra, scaffold or carrier materials for cell transplantation must provide adequate impermeability and function partially as an isolation barrier. To address this challenge, we previously reported the buccal epithelium expanded and encapsulated in scaffold‐hybrid approach to urethral stricture (BEES‐HAUS) technique in animal and clinical studies (Vaddi et al., 2019; Horiguchi et al., 2021; Horiguchi et al., 2023a), in which in vitro–expanded autologous buccal mucosal epithelial cells are encapsulated in a unique nanopolymer scaffold called Festigel (free-from-endotoxin-scaffold of thermoresponsive intelli-gel) and transplanted endoscopically to enhance healing through mucosal coverage. The Festigel, is a sterile, endotoxin-free synthetic thermo-responsive gelation polymer composed of poly (N-isopropylacrylamide-co-n-butyl methacrylate) and polyethylene glycol (PEG) blocks. Festigel is a transparent, non-biological hydrogel that transitions from sol to gel above 20 °C, allowing cells to be suspended at low temperature and forming a stable three-dimensional matrix at body temperature (Kataoka and Huh, 2010; Dedeepiya et al., 2014). The polymer is biocompatible, biodegradable and has selective permeability permitting nutrient and gas exchange. The material’s temperature dependent viscoelasticity (Kataoka and Huh, 2010; Dedeepiya et al., 2014) provides mechanical stability sufficient for endoscopic delivery yet soft enough to conform to urethral mucosa. This scaffold has been tested for cytocompatibility across multiple mammalian cell types including corneal epithelial cells, hepatocytes, chondrocytes, and stem cells with no toxicity or fibroblast overgrowth reported (Kataoka and Huh, 2010; Dedeepiya et al., 2014). Festigel has been used clinically for epithelial cell delivery in periodontal regeneration (Sankaranarayanan et al., 2013) and is used clinically as a scaffold in the BEES-HAUS procedure, approved as per the act on safety of regenerative medicine of Japan (Saiseiiryo, 2025). The Festigel, has been evaluated in both animal and clinical models and has been documented to provide a native-like microenvironment, maintain cellular phenotype, and support engraftment (Horiguchi et al., 2023a). Research on *in vitro* culture of human buccal epithelial cells using Festigel was initially developed for ocular surface diseases, particularly bilateral corneal epithelial disorders (Katoh et al., 2021; Senthilkumar et al., 2023). While Nishida et al. had earlier reported buccal cell–derived epithelial sheets (Nishida et al., 2004), our focus was on developing a contamination-free, xeno-free system avoiding biological substrates such as amniotic membrane and on adapting the methodology for urethral stricture disease. The BEES-HAUS procedure (Vaddi et al., 2019; Horiguchi et al., 2021; Horiguchi et al., 2023a) represents a minimally invasive, endoscopically delivered autologous buccal epithelial cell therapy encapsulated in the Festigel scaffold delivering buccal epithelial cells endoscopically into the urethra rather than relying on invasive open surgical grafting. A potential advantage of BEES-HAUS is that it requires only a small 4–5 mm buccal punch biopsy compared to the larger 50–60 mm grafts harvested for conventional urethroplasty, thereby significantly reducing donor site morbidity. Additionally, because the urethral lumen is preserved during transplantation, the need for surgical manipulation is reduced. The procedure is performed under endoscopic guidance rather than general anesthesia, leading to shorter hospital stays and reduced treatment costs. In our pilot study involving six male patients (Vaddi et al., 2019) with bulbar strictures (2–3.5 cm), autologous buccal cells isolated from a small 4–5 mm mucosal biopsy were expanded *ex vivo* and transplanted after wide endoscopic urethrotomy. Objective outcomes reported include mean peak flow rate (Q-max) of 24 mL/s at 6 months and endoscopic evidence of healthy mucosal continuity at the urethrotomy site. Follow-up of up to 40 months showed recurrence free state in four of six patients, with no graft-site morbidity, infection, or adverse events recorded in any of the patients. Importantly, in the pilot clinical study, the study population included patients with recurrent strictures following multiple surgical reconstructions, urethrotomies, or dilatations as well (Vaddi et al., 2019). Other than the pilot clinical study, supporting preclinical studies in rabbits confirmed engraftment morphologically and histologically. In a reproducible electrocoagulation-induced urethral stricture model, autologous buccal epithelial cells encapsulated in Festigel were transplanted in a transurethral manner. Histology (H&E) demonstrated re-epithelialization with stratified squamous morphology at the repair site (Horiguchi et al., 2021). Immunohistochemistry showed CK14 positivity and GATA-3 negativity at the transplanted region, indicating engraftment of buccal epithelium distinct from native urothelium, while the surrounding urethra remained GATA-3 positive (Horiguchi et al., 2023a). These findings confirmed that the transplanted buccal cells formed a stable epithelial barrier over the urethrotomy site, consistent with the hypothesized mechanism that epithelial coverage mitigates urine-induced fibrosis. Mechanistically, the engraftment of buccal epithelial cells restores epithelial continuity and barrier protection at the urethral lumen, thereby reducing exposure of subepithelial tissues to urine and inflammatory mediators. Although the buccal epithelium retains squamous phenotype (CK14 positive, GATA-3 negative) without full urothelial transdifferentiation (Horiguchi et al., 2021; Horiguchi et al., 2023a), the observed epithelialization is sufficient to prevent fibrotic remodeling, as evidenced by absence of granulation tissue and restoration of luminal patency in both animal and human studies.

### BHES-HAUS: Rationale

Though the BEES-HAUS technique addresses several challenges through hypothesized mechanisms such as providing epithelial coverage, promoting regeneration through transplanted buccal cells that may transdifferentiate into urethral epithelium or through paracrine effects, and utilizing Festigel to create an optimal scaffold environment, certain limitations remain, including the requirement for a 14-day cell culture period, the need for specialized laboratory facilities, and its inherently two-step nature (Foreman et al., 2023). In search of simplified alternatives, Nikolavsky et al. reported a single-step liquid buccal mucosa graft endoscopic urethroplasty, in which mechanically minced buccal mucosal micrografts suspended in fibrin glue were transplanted in both rabbit and clinical models (Scott et al., 2020; Li G. et al., 2024). Building upon this, and inspired by the single-step liquid buccal mucosa graft urethroplasty of Nikolavsky et al. (Scott et al., 2020; Li G. et al., 2024), we developed the BHES-HAUS (buccal epithelium hashed and encapsulated in scaffold-hybrid approach to urethral stricture), a simplified, culture-free modification of BEES-HAUS. In BHES-HAUS, a tiny bit of buccal mucosal biopsy is harvested, defatted and hashed into minute fragments in saline containing antibiotics, then centrifuged to obtain a viable cell-tissue pellet. The pellet is resuspended in sterile, cooled Festigel to form a tissue bits/cells-scaffold mixture. This tissue bits/cells-scaffold mixture is injected endoscopically at the stricture site immediately after a direct visual internal urethrotomy (DVIU). At body temperature, Festigel gels *in situ*, retaining epithelial fragments for engraftment while allowing proliferation and migration across the denuded mucosa. The entire process is performed under aseptic operating-room conditions without need for external laboratory culture. The ongoing prospective clinical trial (CTRI/2025/07/091030) (BHES-HAUS, 2025) evaluates BHES-HAUS for short-segment bulbar urethral strictures. It is a single-arm, open-label study enrolling 15 adult male patients aged above 18 years with recurrent strictures after prior DVIU or dilatation or BMG plasty. Exclusion criteria include multiple strictures, long segment stricture, radiation induced stricture and donor site unavailability (tobacco chewers, submucosal fibrosis, previous oral surgery). Endpoints include (i) primary: restoration of urethral patency measured by peak flow rate (Q-max ≥ 15 mL/s) at post-surgery- 3 weeks, 3 months, 6 months, 12 months) and International Prostate Symptom Score (Pre Surgery, Post Surgery - 3 weeks, 3 months, 6 months, 12 months) (ii) secondary end-points include absence of recurrence at 12 months and absence of adverse events. The study has been registered in the clinical trials registry of India (BHES-HAUS, 2025). The results of this trial will clarify whether this single-sitting, minimally invasive procedure can reproducibly prevent fibrosis and long-term recurrence. It is important to note that the BHES-HAUS can be accomplished in the operating room in the same sitting. Such procedures providing epithelial coverage by cell proliferation and migration within the polymer matrix have been previously demonstrated over a cadaver cornea in which explant tissues embedded in the polymer scaffold was transplanted *in vitro* settings (Senthilkumar et al., 2023). Similar findings have also been documented in pre-clinical and clinical studies of urethral stricture (Vaddi et al., 2019; Horiguchi et al., 2021; Horiguchi et al., 2023a). This single-step, simpler approach is hypothesized to be a feasible alternative for short-segment urethral strictures, particularly as an alternative to dilatation or urethrotomy, by providing epithelial coverage, enhancing local healing and potentially reducing recurrence. The outcomes of the clinical trial which is currently underway (BHES-HAUS, 2025) will be critical to determine whether BHES-HAUS can effectively prevent disease progression and long-term recurrence.

While comparing with the other tissue-engineering techniques reported elsewhere for managing urethral strictures such as acellular biologic matrices (porcine small-intestinal submucosa (SIS) or bladder submucosa; collagen sheets) (Ribeiro-Filho and Sievert, 2015; El-Kassaby et al., 2003; Li et al., 2008; Mantovani et al., 2011), drug-coated balloons (paclitaxel) (Estaphanous et al., 2024; VanDyke et al., 2023), cultured buccal mucosal and minced autologous graft techniques though have shown encouraging short-to mid-term results but each carries limitations relevant to durability, consistency, or invasiveness (Scott et al., 2020; Li G. et al., 2024). Early and long-term experiences with porcine SIS reported satisfactory patency after onlay urethroplasty, including favorable 10-year follow-up cohorts (Ribeiro-Filho and Sievert, 2015; El-Kassaby et al., 2003; Li et al., 2008; Mantovani et al., 2011). However these require open grafting, depend on biologic sourcing, there can be batch to batch variability which can produce remodelling unpredictably (keratinization, contraction etc.) (Ribeiro-Filho and Sievert, 2015; El-Kassaby et al., 2003; Li et al., 2008; Mantovani et al., 2011). Collagen-based inert matrices similarly achieved good 3-year outcomes with histologic epithelialization, but again necessitate open reconstruction and suture-line interfaces are prone to anastomotic narrowing or fistula. Seeding oral keratinocytes onto acellular matrices improved epithelialization, yet this adds *ex vivo* culture complexity and still relies on biologic scaffolds (Ribeiro-Filho and Sievert, 2015; El-Kassaby et al., 2003; Li et al., 2008; Mantovani et al., 2011). Drug-coated balloons (paclitaxel) offer a minimally invasive option with superior 2-year re-intervention-free survival versus standard endoscopy, but do not restore epithelial coverage (Estaphanous et al., 2024; VanDyke et al., 2023). Minced buccal micrografts in fibrin glue as reported by Nikolavsky et al. avoid prolonged culture and show promising success (Scott et al., 2020; Li G. et al., 2024), but depend on biologic adhesives. Similar approach of minced buccal tissue has been reported clinically by another team (Singh et al., 2025; Gaur et al., 2025) but their methodology is different from Nikolavsky et al., as they report transplantation of the supernatant after centrifugation with fibrin glue, excluding large tissue fragments and the disadvantage is also in the use of fibrin glue (Vaddi et al., 2025). Another cell-therapy approach of *in vitro* cultured buccal cells has been reported clinically (Kulkarni et al., 2021) however this method has not been proven with proof of cellular engraftment clinically or with any pre-clinical evidence to our knowledge. One more tissue engineering product, Mukocell has been reported but it again has the disadvantage of need of *in vitro* culture (Karapanos et al., 2021). In contrast, Festigel is fully synthetic, endotoxin-free and xeno-free, forming an *in situ* 3D matrix at body temperature that retains epithelial tissue bits or cells at the urethrotomy while allowing nutrient diffusion. This minimizes urine contact with the wound bed during early healing, reduces fibro-inflammatory exposure, and eliminates the batch to batch variability and contamination risks of biologic scaffolds (Vaddi et al., 2019; Horiguchi et al., 2021; Horiguchi et al., 2023a; Kataoka and Huh, 2010; Dedeepiya et al., 2014; Sankaranarayanan et al., 2013). BHES-HAUS helps in restoration of autologous epithelium endoscopically with a small oral biopsy and does not require a culture period thereby aligning minimal invasiveness with epithelial barrier restoration.

Although early clinical and preclinical results of BEES-HAUS have demonstrated safety and feasibility (Vaddi et al., 2019), potential procedure-related risks of BEES-HAUS and BHES-HAUS merit consideration. Graft infection is a theoretical concern due to the implantation of an epithelial cell–scaffold composite into a urinary environment. However, in both the pilot human study and ongoing clinical trial, strict asepsis is maintained wherein the buccal harvest and urethrotomy are performed under sterile conditions, and the Festigel carrier is endotoxin-free and non-biologic reducing risk of contamination. No postoperative infection or fever was reported in our pilot clinical study of BEES-HAUS (Vaddi et al., 2019). Graft displacement or washout could occur if the scaffold does not adequately adhere to the urethrotomy base. Festigel’s thermo-responsive transition at body temperature forms a soft gel that conforms to the mucosal defect and retains cells *in situ* until early epithelial anchorage occurs which has been proven prior in ocular surface and urethral surface in animal studies (Horiguchi et al., 2021; Horiguchi et al., 2023a; Dedeepiya et al., 2014; Senthilkumar et al., 2023). Urethral obstruction due to scaffold bulk is unlikely because Festigel’s temperature-responsive viscoelasticity (Kataoka and Huh, 2010; Dedeepiya et al., 2014), allows the gel to spread rather than blocking as an obstructive plug. Endoscopic visualization during injection ensures even distribution along the lumen. Also, in terms of affinity or adherence to the cellular surface for the required period of time and then be washed away instead of blocking the passage or retention, we have earlier proven by washing of fluorescent coated Festigel (Senthilkumar et al., 2023). In both animal and clinical studies, no obstruction or flow reduction was observed because of the Festigel, as the catheter was retained for 21 days allowing time for engraftment of cells following which the gel gets washed away with the flow of urine, as evidenced in clinical and pre-clinical studies (Vaddi et al., 2019; Horiguchi et al., 2021; Horiguchi et al., 2023a). Donor-site morbidity from a very small buccal punch is minimal compared to conventional graft urethroplasty, where 50–60 mm mucosal strips are harvested. Overall, while monitoring for infection, displacement, or temporary irritative symptoms at donor site remains necessary, the synthetic xeno-free scaffold Festigel, minimal harvest size, and endoscopic delivery together mitigate the common complications associated with open grafting and biologic materials. Thus, the BEES-HAUS procedure combining 2D cultured cells with fibroblast morphology secreting IGF-1 and 3D Festigel cultured cells growing with epithelial morphology (Horiguchi et al., 2023b), stands superior for its dual advantages of the former group of cells yielding paracrine effect aiding healing while the later engrafting and providing epithelial coverage compared to other techniques and conventional procedures for treating urethral strictures. Further research into value adding to the simplified single-step BHES-HAUS is essential based on outcome in translational and clinical studies in future enable it be included in the guidelines for management of urethral stricture disease.
